# Effects of Three Different Injection-Molding Methods on the Mechanical Properties and Electrical Conductivity of Carbon Nanotube/Polyethylene/Polyamide 6 Nanocomposite

**DOI:** 10.3390/polym11111779

**Published:** 2019-10-30

**Authors:** Dashan Mi, Zhongguo Zhao, Wenli Zhu

**Affiliations:** 1 Department of Mechanical Engineering, Hubei University of Arts and Science, Xiangyang 441053, China; 2 School of Polymer Science and Engineering, Shaanxi University of Technology, Hanzhong 723001, China; zhaozhongguo@snut.edu.cn

**Keywords:** electrical conductivity, mechanical properties, nanocomposites, injection molding

## Abstract

Morphological evolution under shear, during different injection processes, is an important issue in the phase morphology control, electrical conductivity, and physical properties of immiscible polymer blends. In the current work, conductive nanocomposites were produced through three different injection-molding methods, namely, conventional injection molding, multi-flow vibration injection molding (MFVIM), and pressure vibration injection molding (PVIM). Carbon nanotubes in the polyamide (PA) phase and the morphology of the PA phase were controlled by various injection methods. For MFVIM, multi-flows provided consistently stable shear forces, and mechanical properties were considerably improved after the application of high shear stress. Shear forces improved electrical property along the flow direction by forming an oriented conductive path. However, shear does not always promote the formation of conductive paths. Oscillatory shear stress from a vibration system of PVIM can tear a conductive path, thereby reducing electrical conductivity by six orders of magnitude. Although unstable high shear forces can greatly improve mechanical properties compared with the conventional injection molding (CIM) sample, oscillatory shear stress increases the dispersion of the PA phase. These interesting results provide insights into the production of nanocomposites with high mechanical properties and suitable electrical conductivity by efficient injection molding.

## 1. Introduction

Nanocomposites are attracting considerable interest in applied and fundamental research [1]. Carbon nanotubes (CNTs) are representative examples of nanofillers. They have exceptional aspect ratios, elastic moduli, strength, electrical properties, thermal conductivity, and chemical stability. Although CNTs have satisfactory mechanical properties, only small fractions of their stiffness and strength are translated into a matrix, in which CNTs are embedded by aggregation, twisting, and curling [2]. Thus, critical issues relating to the enhancement of the mechanical properties of CNT composites are the disaggregation and control of the dispersion of CNTs in polymers. Regarding electrical properties, well-dispersed nanotubes are efficiently interconnected and thus decrease the amount of fillers for electrical percolation [3,4,5]. Other studies demonstrated that the poor dispersion of nanotubes in a matrix results in the formation of agglomerates and significantly increases the percolation threshold [6].

In immiscible polymer blends, the selective localization of a conductive filler in any phase or at an interface can improve electrical conductivity at low filler concentrations [7]. A low percolation threshold is ideal for maintaining the rheological, physical, and mechanical properties of materials and the economic competitiveness of expensive conductive fillers, such as CNTs [8]. Many factors, such as mixing duration, mixing sequence, affinity of CNTs to each component, and the viscosity ratio between two components, and interfacial tension, influence the selective location of CNTs in polymer blend composites [9].

High-density polyethylene (HDPE) has low permeability to water vapor, and polyamide (PA) has low oxygen permeability. Therefore, the blends of HDPE and PA can serve as good barriers for water and oxygen and can improve the mechanical properties of HDPE [10]. However, HDPE and PA are incompatible, and CNTs are usually located in the PA phase, regardless of mixing sequence [7,11]. An ideal PA morphology can improve the conductivity and mechanical properties of nanocomposites. PA morphology is influenced by interfacial tension, nanoparticle content, and shear rate [12,13].

A strong shear rate can form a molecular orientation structure (such as shish kebab or shish-kebab-like cylindrulite), which is regarded as a kind of self-reinforced structure [14,15,16]. Shear can influence the orientation of CNTs and may increase electrical conductivity at one direction and at low nanotube loading [17]. However, shear stress can disperse CNTs and destroy the contact between CNT clusters and can thus reduce conductivity [18].

Some unusual injection-molding technologies have been developed, such as shear-controlled orientation in injection molding (an injection-molding machine equipped with a double live-feed molding device and used for producing bars; the piston pressure is not constant but has periodical variation) [19,20,21] and rotation, compression, and expansion molding (a center-gated disk is produced under external mechanical loads; cavity molding surfaces rotate and move axially in either a steady or oscillating mode) [22,23]. These technologies have been developed to impose strong shear fields on polymer melts. Various injection methods provide different melt-flow modes and shear rates that control the phase morphology of nanocomposites.

**Figure 1:**
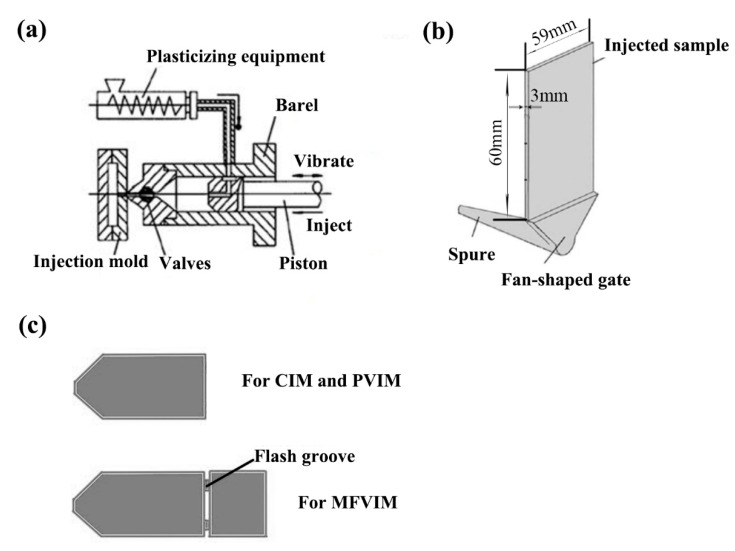
Schematic representation of (**a**) injection-molding machine, (**b**) specimens, and (**c**) mold design for CIM, PVIM, and MFVIM.

In the present work, conventional injection molding (CIM), multi-flow vibration injection molding (MFVIM) [24], and pressure vibration injection molding (PVIM) [25,26] were used in the preparation of HDPE/PA/CNT nanocomposites. Figure 1 shows the schematic illustration of the mold and machine. In PVIM, a vibration system is used in addition to an injection system. The former provides oscillatory pressure, whereas the latter provides basic pressure. During the packing stage, a periodically changing pressure acts on the melt in the runner and mold cavity until the injection gate freezes. MFVIM is a new process of PVIM. In contrast to PVIM, MFVIM uses a mold with a flash groove that can form multi-flows in a sample during the packing stage. Four melt flows are used to generate a stable high shear rate for MFVIM. An unstable high shear stress for PVIM is achieved by introducing oscillatory pressure.

Shear rate can improve mechanical properties; however, various shear methods produce nanocomposites with varying phase morphology, which influences conductivity. Regarding injection-molding methods, achieving a balance between mechanical and conductive properties remains a challenge. This study aims to investigate the influence of all composing aspects—three different injection methods and various PA and CNT contents—on the mechanical and conductive properties of HDPE/PA/CNT nanocomposites. This study intends to explore the effects of stable and unstable shears on the electrical conductivity of the injection-molded samples and to determine a method for improving the conductive and mechanical properties simultaneously.

## 2. Experimental Section

### 2.1. Materials and Sample Preparation

CNTs with a diameter and length of approximately 9.5 nm and 1.5 μm, respectively, were purchased from Nanocyl SA (Nanocyl, Sambreville, Belgium) and used without any pretreatment. HDPE (5000S) was obtained from Daqing Petroleum Chemical Co. (Daqing, China). The average molecular weight (Mw) was 5.28 × 10^5^ g/mol, and the melt flow index (MFI) was 0.9 g/10 min (190 °C, 2.16 kg). PA6 (M2800) was purchased from Guangdong Xinhui Meida Nylon Co., Ltd. (Jiangmen, China). Its melting temperature and MFI were 220 °C and 11 g/10 min (300 °C, 2.16 kg), respectively. PA was dried in an air-circulating oven for 8 h, at 90 °C, prior to mixing. HDPE, PA, and MWCNT were mixed in a twin-screw extruder (SHJ-25 corotating twin-screw extruder with L/D ratio of the screws was 32, and D is 25 mm), at a rotational speed of 100 rpm and barrel temperature of 250 °C. Good CNT dispersion was achieved with a two-step process. The CNTs were first melt-mixed with HDPE, to form a master batch containing 10 Phr CNTs. The master batch was then further melt-compounded with pure HDPE and PA. The final nanocomposites contained 0.5, 1, and 2 Phr CNTs, and the PA content was 50 or 25 wt.%. The subsequently extruded compound was cooled down to room temperature in a water bath and granulated.

As shown in Figure 1, the samples prepared using different injection methods exhibited similar shapes and sizes. The same mold was used in CIM and PVIM, but a mold containing two flash grooves was used in MFVIM. CIM, MFVIM, and PVIM were used in the preparation of 59 mm × 60 mm × 3 mm rectangular samples. The samples were cut into certain shapes (dumbbell or strip), along the flow direction, during mechanical tests.

**Figure 2:**
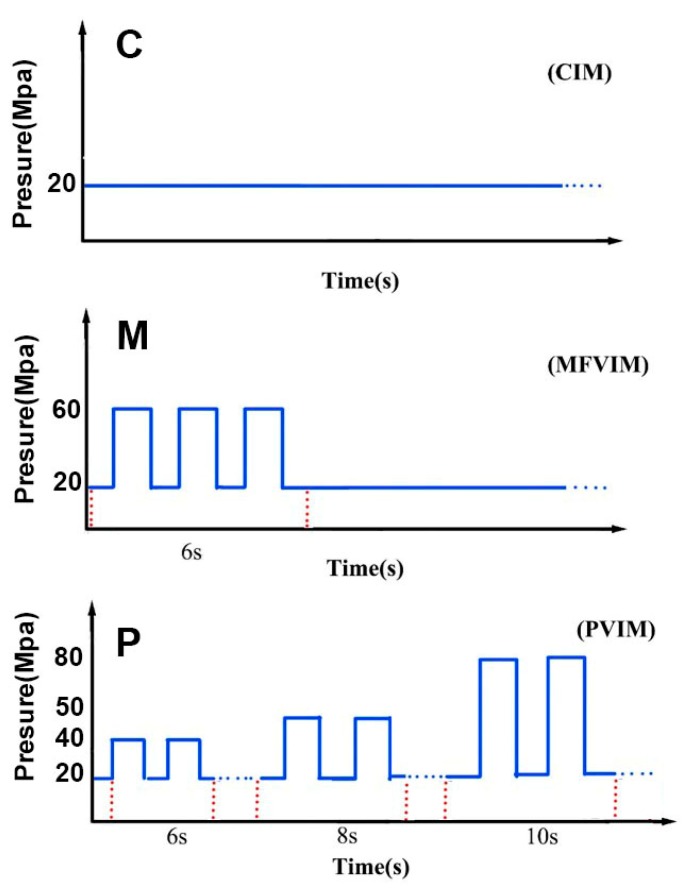
Schematic diagram of the pressure changes in different injection-molding methods.

CIM: Samples were prepared by CIM at a barrel temperature of 250 °C, mold temperature of 60 °C, and injection setting pressure of 20 MPa. Figure 1 and Figure 2 show that the melt filled the cavity under a constant setting pressure, indicating that the maximum melt pressure was unchanged.

**Figure 3:**
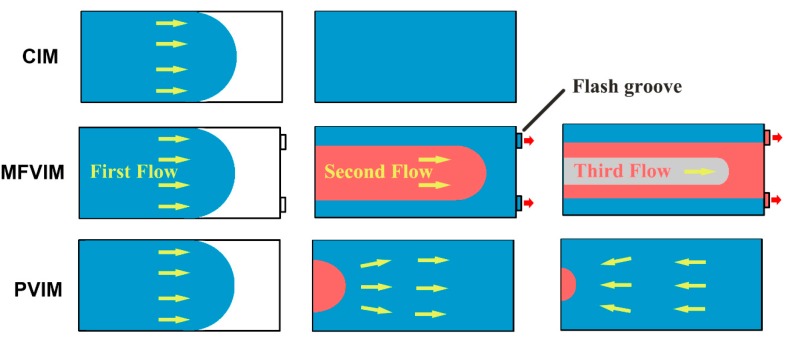
Schematic melt flow in different injection-molding cavities. The arrows indicate the melt-flow direction. The different colors represent the melts that enter the die at different times. Three melt flows are drawn in MFVIM.

MFVIM: Samples were prepared at a barrel temperature of 250 °C and mold temperature of 60 °C. The mold was initially filled with melt at an injection pressure of 20 MPa, and no melt spilled through the flash groove. As shown in Figure 2 and Figure 3, oscillatory pressure (60 MPa) was subsequently introduced for the formation of a second/third flow, during the packing stage, and a part of the melt could be pushed out of the cavity through the flash groove. Each flow would increase the shear area by creating a new shear layer. Figure 3 shows only three melt flows to simply express the principle. In fact, four flows were achieved in the experiment by adjusting vibration frequency. The vibration was performed for approximately 6 s. MFVIM provides high shear stress for the formation of highly oriented PP crystal structures, such as shish kebab [14,24].

PVIM: Samples were prepared at a barrel temperature of 250 °C and mold temperature of 60 °C. The injection piston was driven by a vibration system that provided oscillatory pressure and an injection system that provided basic pressure. No flash groove appeared in the mold cavity, unlike in MFVIM. Thus, oscillatory pressure during the packing stage caused periodical compression and decompression on the nanocomposite melt, and periodical shearing occurred at the melt-solid interface (Figure 3). The pressure drop in the melt increased in the freezing layer in the runner. Additional pressure was needed for melt vibration. Therefore, sufficient shear stress in the cavity was achieved by gradually increasing pressure from 40 to 80 MPa (Figure 2). Notably, periodical shearing at the melt-solid interface can lead to local stick-slip dynamics [27] and disturb polymer ordering in the vicinity of metallic walls [28]. However, when melt flows into a mold, melt in the outer layer comes into contact with the cold wall of the mold (60 °C) and solidifies in the first second. Therefore, subsequent vibration pressure has no effect on the surfaces of the workpieces. Hence, although CIM, MFVIM, and PVIM provide different injection processes, their products exhibit similar surface qualities.

The sample designation and compositions are provided in Table 1. A and B represent the PA content; 0.5, 1, and 2 represent CNT content; C, M, and P represent CIM, MFVIM, and PVIM, respectively. For example, A0.5(C) represents the sample with PA content of 50 wt.%, molded by CIM, and the CNT content is 0.5 Phr.

### 2.2. Optical Microscope Observations (POM)

For optical microscopy, thin slice sections, nearly 5 microns in thickness, were prepared by cutting at room temperature, using an RM 2016 microtome (Leica Instruments Ltd, Leica, Germany). The specimens were investigated by applying to Motic BA300 optical microscope system (BA300, Motic China Group Co., LTD., Xiamen, China).

### 2.3. Scanning Electron Microscope (SEM)

A Hitachi scanning electron microscope (Model S4800; Hitachi, Osaka, Japan) was used for examining the morphological variation of PA and HDPE phase, as well as the CNT in the interface. Samples were etched by formic acid at 60 °C, for 6 h. Prior to microscopic examination, the surfaces of the specimens were coated with a thin layer of gold by ion sputtering.

### 2.4. Measurement of Mechanical Properties 

The tensile test was conducted at room temperature (20 °C) on an electro-universal testing machine (Instron 5569, Instron Corp., Norwood, MA, USA) with a cross-head speed of 30 mm/min. The notched Izod impact strength of the specimens was measured with a XJUD-5.5 Izod machine (Chengde yonghao Corp., Chengde, China), at room temperature. Before the test, a 45° V-shaped notch (depth 2 mm) was made. All of these tests were conducted along the injection direction, and the values of all the mechanical properties were calculated as averages of over five samples.

### 2.5. Electrical Properties Measurements

DC measurements:

The conductivity of samples along the flow direction were performed at room temperature, on specimens, using a Keithley 6487 source meter (Tektronix Corp., Bracknell, Berkshire, United Kingdom). Both ends of the specimens were coated with conductive silver paint. The electrical conductivity *σ* was then calculated according to Equation (1):

*σ* = *L*/*RA* (1)

where *L* is the distance between the electrodes, *A* is the cross-sectional area, and *R* is the measured resistance.

## 3. Results and Discussion

### 3.1. Mechanical and Electrical Conductivity 

**Figure 4:**
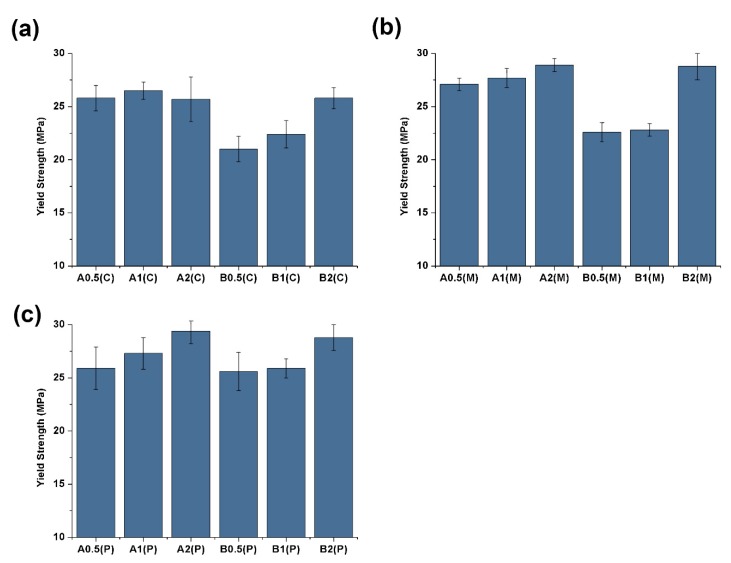
Yield strength of (**a**) CIM, (**b**) MFVIM, and (**c**) PVIM.

One of the advantages of CNTs as reinforcement fillers is their large surface areas, which can induce good adhesion with polymeric matrices [29,30]. However, this feature often leads to strong aggregation by Van der Waals attraction [31,32,33]. Hence, the degree of CNT dispersion can greatly influence the final mechanical properties. As shown in Figure 4, increase in CNT content improves tensile properties, except in A2(C). The stress-strain curves can be found in Support Information (Figure S1). The tensile strength of B2(C) is 23% higher than that of B0.5(C). The tensile strengths of A2(M), B2(M), A2(P), and B2(P) are higher than those of A0.5(M), B0.5(M), A0.5(P), and B0.5(P) by approximately 7%, 27%, 14%, and 13%, respectively. Bing et al. [31] reported that increases in the volume fractions of CNTs enhance the alignment of well-dispersed composites when volume fractions are small (<9.4%). In addition, unlike in CIM, a high shear stress improves the dispersion of CNTs in PVIM and MFVIM. The good dispersion and preferential orientation of MWNT bundles can be achieved by dynamic packing injection molding [29]. Improved dispersion affords PVIM- and MFVIM-molded samples high yield strength. For example, the yield strength of B2(M) and B2(P) is 28.8 MPa, which is greater than that of B2(C), at 25.8 MPa.

PA6 demonstrates better mechanical properties than HDPE and is widely used as an engineering plastic. Thus, high PA content can increase tensile strength. For example, the yield strength of A0.5(C) (25.8 MPa) with 50% PA content is higher than that of B0.5(C) (21.0 MPa), which has a PA content of 25%. However, the CNTs show effective enhancement in composites with low PA contents because high CNT concentrations in low-PA-content composites can help improve CNT networks [13]. Thus, A2(C), A2(M), and A2(P) obtained similar tensile strengths than those of B2(C), B2(M), and B2(P), respectively, regardless of PA content.

**Figure 5:**
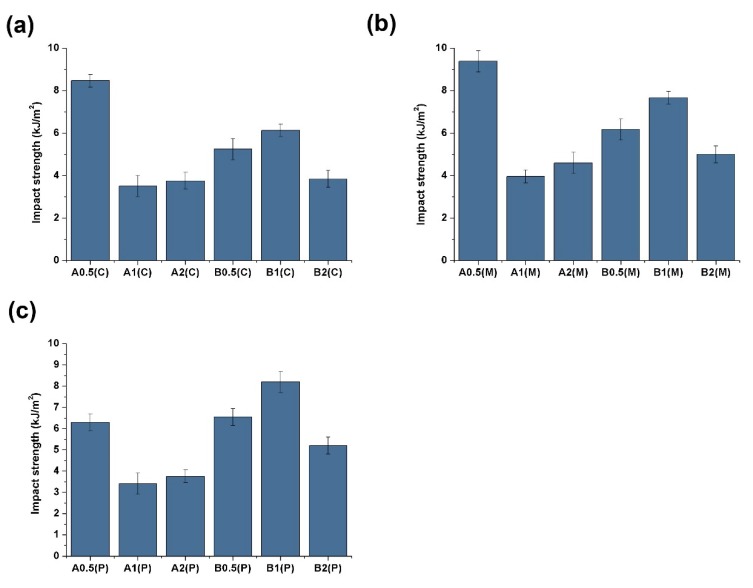
Impact strength of different samples molded by (**a**) CIM, (**b**) MFVIM, and (**c**) PVIM.

The impact strength of the samples is shown in Figure 5. The CIM sample has the lowest CNT (0.5 Phr) content, as shown in Figure 5a, but has the highest impact strength of 8.47 kJ/m^2^. CNT (1 Phr) reduces the impact strength to 3.5 kJ/m^2^. In the PVIM and MFVIM samples (Figure 5b,c), high CNT content reduces impact strength by approximately 50%, when the PA content is 50%. However, the CNT content of 1 Phr always shows the highest impact strength, even at various injection methods, when the PA content is 25%.

**Figure 6:**
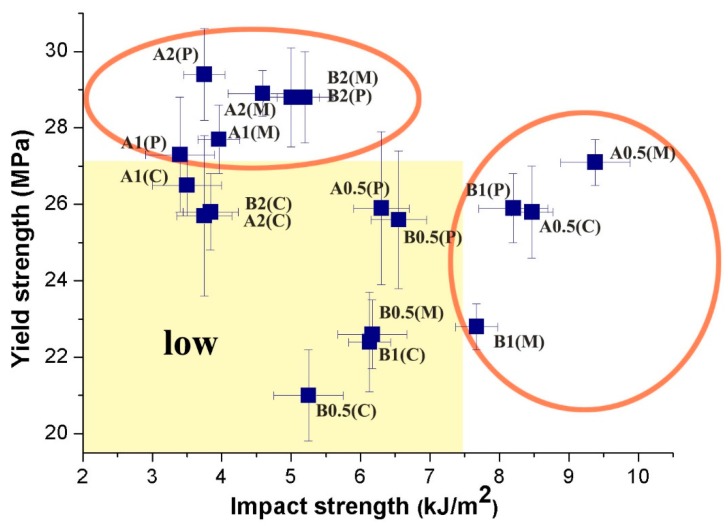
Impact strength versus yield strength. The yellow box represents the samples with low tensile and impact strength, and the high-performance samples are circled.

The tensile and impact properties of the samples are shown in Figure 6. The effects of various parameters on the mechanical properties of the product were compared. The properties of CIM samples are primarily concentrated in the low region. For example, the impact and tensile properties of B0.5(C) are only 5.25 kJ/m^2^ and 21 MPa, respectively. As CNT content increases to 2 Phr (B2(C)), tensile properties increase from 21 to 25.8 MPa. This phenomenon is similar to that reported by other studies, suggesting that CNTs can reinforce nylon-6 composites and nylon-6/polypropylene composites, without any treatment [34,35]. However, adding CNTs reduces impact strength, especially that of the CIM sample. The impact strength of B2(C) decreases by approximately 27% relative to that of B0.5(C). Many studies reported a reduction in toughness after the incorporation of CNTs, even at low loadings [36,37]. Even with the use of other nanoscale fillers, conventional toughening mechanisms, such as crack pinning and crack deflection, cannot be directly transferred to polymer nanocomposites [38].

Overall, the samples processed by MFVIM and PVIM possess better mechanical properties than the CIM samples. The yield of B2(M) is 28.8 MPa, and its impact strength is 5.0 kJ/m^2^, which are 10% and 30% higher than those of B2(C), respectively. The improved properties may be primarily attributed to the high mechanical performance of the oriented crystals [26,39,40]. A high shear stress orients the CNTs or CNT aggregates. In polymer/CNT nanocomposites, the orientation of nanotubes can improve toughness because of crack-wake bridging, that is, when the nanotubes are oriented normally to the craze/crack growth direction [1].

**Figure 7:**
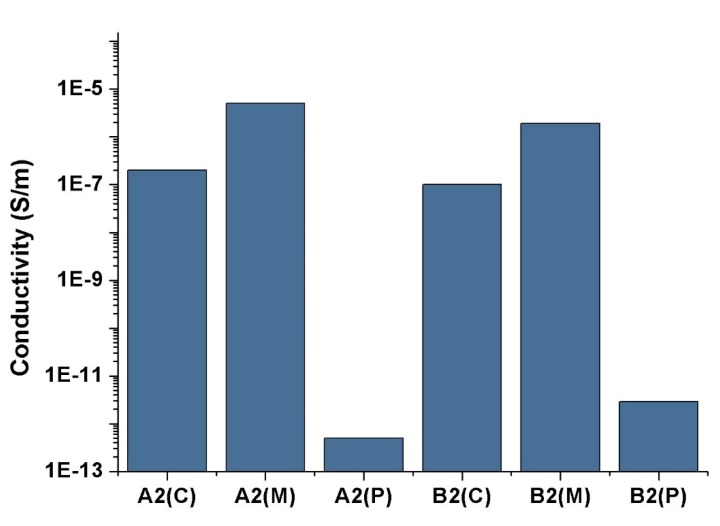
The electrical conductivity of nanocomposites with 2 Phr CNT.

The results of the electrical conductivity test are shown in Figure 7. A2(C) and B2(C) exhibit similar conductivity of approximately 2.0 × 10^−7^ S/m and 1.0 × 10^−7^ S/m. The A2(M) sample has the highest conductivity of approximately 5.1 × 10^−6^ S/m. The conductivity value of B2(M) is 1.9 × 10^−6^ S/m, which is slightly lower than that of A2(M). Thus, the MFVIM can enhance the conductivity of HDPE/PA/CNT nanocomposites. The conductivity of the PVIM samples is considerably lower than that of the CIM or MFVIM samples. The conductivity of A2(P) is only 5.0 × 10^−13^ S/m, and that of B2(P) is only 2.9 × 10^−12^ S/m. These values are considerably lower than those observed in the polymer/CNT nanocomposites. For example, the polypropylene/CNT nanocomposite, the electrical conductivity of the samples prepared by PVIM is three orders of magnitude higher than that of the samples prepared by CIM at the same CNT concentration (3 wt.%) [41].

The electrical conductivity of the nanocomposites is related to the location of CNTs and phase morphology. In the experiment, CNT was first mixed with HDPE to form the masterbatch, which was then blended with PA and HDPE. When a carbon filler is pre-compounded in the thermodynamically unfavored polymer phase, it usually migrates toward the favored phase during melt mixing [29]. For example, CNTs migrate from poly(styrene-co-acrylonitrile) to polycarbonate (PC) [42], from HDPE to PC [43], and from poly(lactic acid) to poly (e-caprolactone) [44]. In the present work, CNTs are selectively localized in the PA phase in the HDPE/PA/CNT blends [11]. Sumita’s model is widely used in predicting the distribution of fillers from a thermodynamic viewpoint and involves the calculation of the wetting coefficient *ω*_a_ based on Young’s Equation [45,46]:

(2) $$\omega_{a}=\frac{\gamma_{A/CNT}-\gamma_{B/CNT}}{\gamma_{A/B}}$$

where $\gamma_{A/CNT}\;$ is the interfacial tension between component A and CNTs, $\gamma_{B/CNT\;}\;$ is the interfacial tension between component B and MWCNTs, and $\gamma_{A/B}$ is the interfacial tension between components A and B. When *ω*_a_ > 1, the MWCNTs are located in the B phase; when *ω*_a_ < −1, MWCNTs are distributed into the A phase; and when −1 < *ω*_a_ < 1, MWCNTs are confined at the interface. The geometric-mean equation is commonly used in the calculation of interfacial energy $\gamma_{A/B}$ between polymer pairs [47]:

(3) $$\gamma_{A/B}=\gamma_{A}+\gamma_{B}-2\left(\sqrt{\gamma_{A}^{d}\gamma_{B}^{d}}+\sqrt{\gamma_{A}^{p}\gamma_{B}^{p}}\right)$$

where $\gamma_{A}$ and $\gamma_{B}$ are the surface energy of components A and B (as shown in Table 2), respectively; $\gamma_{A}^{d}$ and $\gamma_{B}^{d}$ are the dispersed parts of the surface energy of components A and B, respectively; and $\gamma_{A}^{p}\;\mathrm{and}\;\gamma_{B}^{p}\;\mathrm{are}\;\mathrm{the}\;$ polar parts of the surface energy of components A and B, respectively.

The calculated wetting coefficients, obtained after polymers A and B were set as PE and PA, respectively, are negative values (−2.32), lower than −1. This finding indicates that an interfacial energy drove CNT from PE to PA. Thus, conductivity was controlled on the basis of PA phase morphology. The filler quantity at which the conductive path was realized was called the “percolation threshold,” which has been the subject of considerable research. Turning PA6 into a conductive polymer requires a relatively high threshold due to the good interactions of CNTs with the matrices. CNTs are probably wrapped by the PA6 matrix, and electron conduction capacity is further reduced. Aref et al. showed that the addition of 3 vol% CNTs into PA6 can cause the near-zero slope of a G’, indicating a full-volume-spanning network of tubes [48]. Zonder et al. indicated that the rheological and electrical percolation threshold of PA6/ABS/CNT (50/50/x) are 1–2 and 3–4 wt.%, respectively [50]. Steinmann found that the electrical percolation threshold is around 5 wt.% for PA6/CNT.

Given that the CNTs are selectively located in the PA phase, the density of CNTs in A2(C/M/P) and B2(C/M/P) should be 4 wt.% and 8 wt.%, respectively. However, the density of CNTs is not the only factor affecting conductivity. For example, samples A2(C) and B2(C) have the same electrical conductivity. The morphology of the PA phase can greatly influence the conductivity.

### 3.2. Phase Morphology

**Figure 8:**
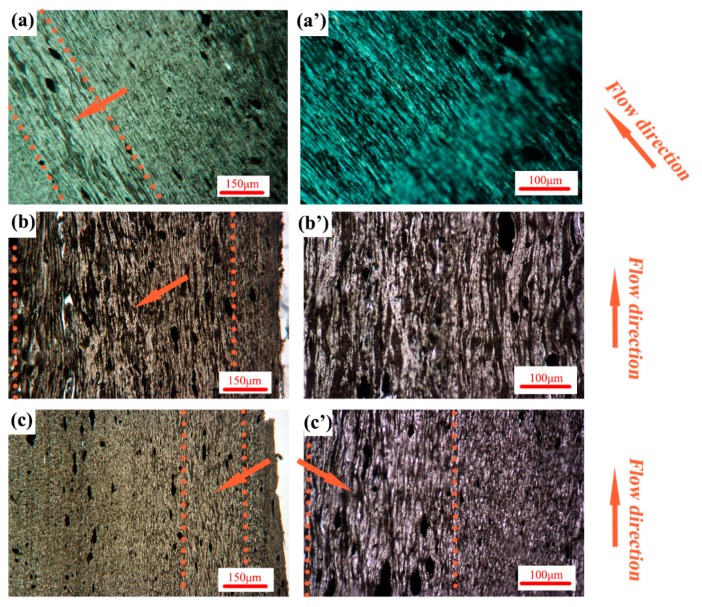
POM images of A2(C) (**a**,**a**’); A2(M) (**b**,**b**’); and A2(P) (**c**,**c**’).

The POM images of A2(C/M/P) are shown in Figure 8. The CNTs show selective distribution in the PA phase. The phase morphology of the PA phase can be easily valuated by observing the black stripe. Gradient morphology is developed by various injection methods and is affected by processing conditions [51,52]. As shown in Figure 8a, a thin shear layer (shear area marked with dotted lines and arrow) appears in A2(C). The PA phase in the shear layer can form conductive pathways. Outside the shear layer, the PA phase is still oriented along the flow direction (Figure 8a’) by a low shear strength. The shear layer in the MFVIM sample (Figure 8b) is considerably thicker than that in the CIM sample (shear area marked with dotted lines and arrow); thus, conductive pathways are abundant, which increase electrical performance to an order of magnitude. The PVIM sample has a shear layer similar to that of CIM (shear area marked with dotted lines and arrow), which forms by first entering melt flow during molding. Given the early freezing time, subsequent vibration pressure has no effect on shear-layer morphology. Therefore, conductive paths are observable in the PVIM samples, as shown in Figure 8c,c’. However, periodical shearing stress in the inner layer shreds the pathways and forms isolated PA phase (indicated by the arrow in Figure 8c’), thereby hindering the passage of electrons and increasing resistance. This occurrence further demonstrates that the dispersed phase can be deformed into a layered-like structure when interfacial tension, the viscosity ratio, and melt elasticity are relatively small. Although some of these values are relatively large, dispersed droplets are not easily deformed and form ellipsoidal or fiber-like structures [53]. Many studies have focused on the effects of nanoparticles on the final morphology and rheological, mechanical, and electrical properties of blends [54]. The results show that morphological formation is largely dependent on the distribution of nanoparticles within blends [55]. A previous study showed that the viscosity of PA is considerably lower than that of HDPE at 250 °C [11]. The rheological behavior of PE is practically unaffected by CNT addition of up to 2 wt.%, whereas that of PA is sensitive to CNT addition; at the low-frequency region, the dynamic storage modulus gradually increases with CNT loading. The different rheological responses of the polymers to CNT addition are attributed to the superior interaction of the PA chains with CNTs [7]. Gooneie et al. found that the addition of CNTs to PA6 droplets results in the development of solid-like elastic structures within droplets. At increasing CNT content, droplets tend to maintain their shapes [13]. In addition, droplet retraction kinetics is evident due to the formed CNT networks. At high shear rates, relaxation slows down [56]. Generally, high shear stress stretches the PA phase into a fiber-like structure and forms conductive pathways. Increased stable shear stress provided by MFVIM can widen the area of a fiber-like PA phase. However, periodical shear relaxes the PA phase into dispersed droplets.

**Figure 9:**
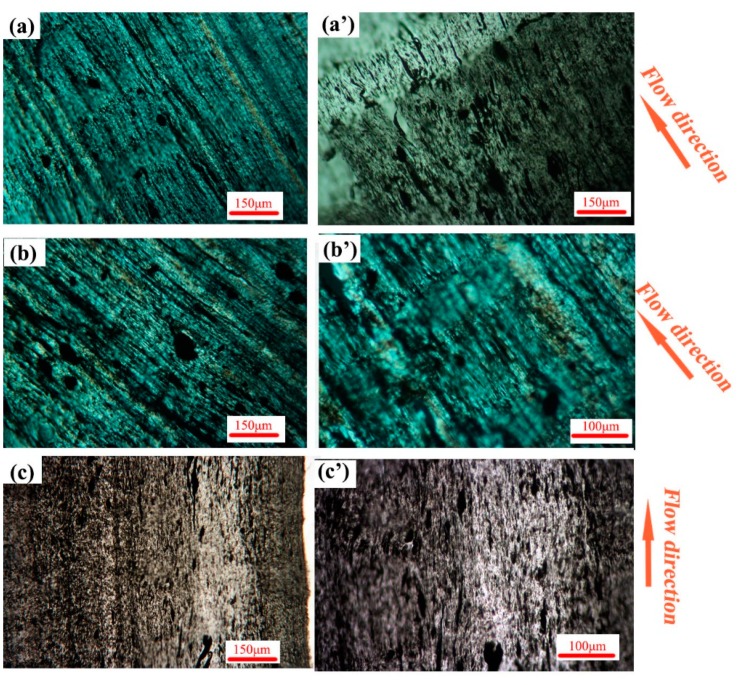
POM images of B2(C) (**a**,**a**’); B2(M) (**b**,**b**’); and B2(P) (**c,c**’).

When the PA content drops to 20 wt.% in the MFVIM and CIM samples, the elongated PA phase, which forms a conductive path, remains observable (Figure 9). However, the shear layer is difficult to distinguish at a low PA content and therefore not marked. In the PVIM samples, the PA phase in B2(P) is more fragmented than that in A2(P), and forming a complete conductive path is difficult. 

**Figure 10:**
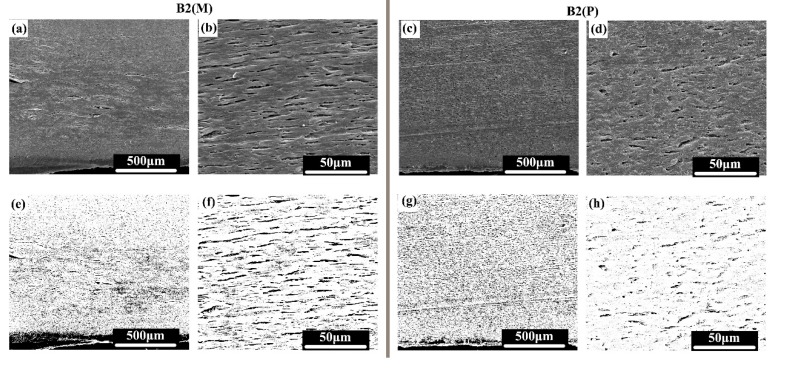
Phase morphology of the immiscible blend. The first row presents the original SEM photo, whereas the second row shows the modified picture for clear observation. Flow direction is horizontal. (**a**,**b**,**e**,**f**) Phase morphology of B2(M); and (**c**,**d**,**g**,**h**) phase morphology of B2(P).

The PA phase was etched by formic acid so that the PA phase could be clearly observed by SEM. The melt-flow direction is horizontal (Figure 10). The first row is the original SEM photo, and the second row is the modified high-contrast image. When the scale of the MFVIM sample (B2(M)) is 500 µm, only diffused black points can be observed. At the magnification scale of 50 µm, a large number of oriented gullies can be observed along the melt-flow direction. The width of the gully is only approximately 1 µm, but the length is several tens of micrometers. However, although some PA phases are oriented along the flow direction in the PVIM sample(B2(P)), the gullies are not connected, and abundant dispersive PA phases are present. This finding indicates that generated multiple flows in the MFVIM contribute to the formation of a long and oriented PA phase and result in high conductivity. However, shearing force caused by vibration pressure increases the dispersion of the PA phase in the PVIM sample. The good dispersion of the PA phase prevents the formation of conductive pathways because of the dispersion of CNTs in the PA phases.

**Figure 11:**
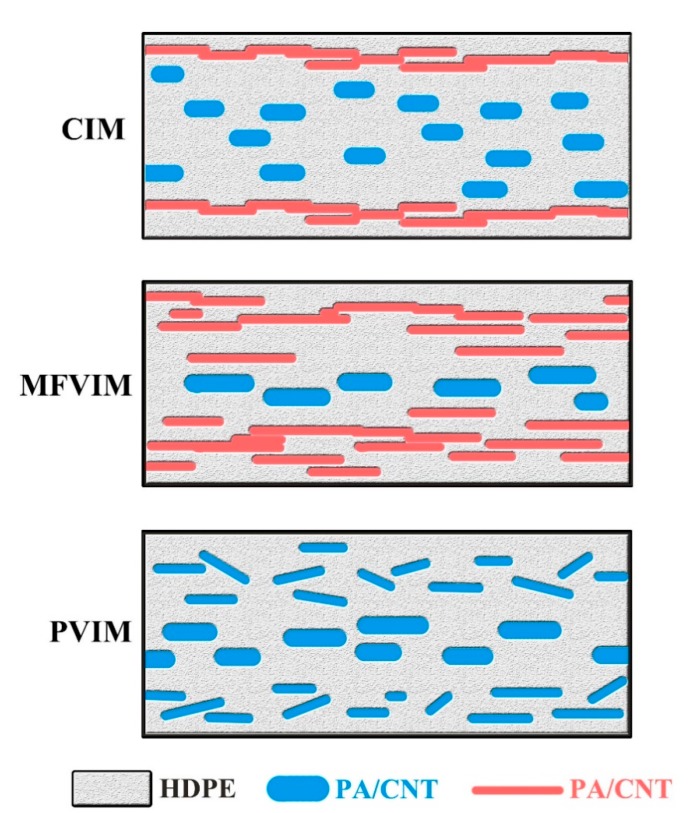
Relationship between conductivity and phase morphology. The blue lines indicate the insulated PA/CNT, whereas the red lines represent the PA/CNT that forms the conductive path.

The relationship between conductivity and phase morphology is shown in Figure 11. The sample prepared by CIM shows a typical skin–core structure, and an oriented conductive path can be formed within the shear layer. However, shear stress in the core layer is weak. Although an oriented PA phase is formed by a weak shear, a perfect conductive path cannot be formed. In the MFVIM sample, the shear layer progressively thickens after the introduction of a multi-flow, which can provide enough shear stress for the formation of a conductive path. Hence, the number of conductive paths formed through MFVIM is higher than that through CIM, and conductivity can be increased by an order of magnitude. In PVIM, when pressure is at its peak value, the melt processed is pressed into the mold cavity, but the melt is discharged from the cavity when the pressure is at the valley value. Thus, periodical shear is generated repeatedly. Periodic shear can considerably improve the mechanical properties of the PVIM samples by forming orientation structures. However, periodical shear is not always in the same direction. When parts cool, the direction of shear force changes. Shear force destroys conductive paths and reduces the conductivity of the PVIM sample by six orders of magnitude compared with that of the CIM sample. In general, the CIM method remains the most versatile choice; MFVIM can help further improve the electrical conductivity and mechanical properties; PVIM produces insulating nanocomposites with high mechanical properties.

## 4. Conclusions

HDPE/PA/CNT nanocomposite was prepared through three injection methods. Compared with the CIM sample, the MFVIM sample can provide a stable multi-flow and form a wide shear layer. PVIM can provide unstable oscillatory pressure and thereby cause periodical compression and decompression on a nanocomposite melt. At low CNT content (0.5 wt.%), MFVIM can be used to obtain an outstanding impact strength of 9.38 kJ/m^2^. At a high CNT content, PVIM can be used to obtain an outstanding tensile strength of 29.4 MPa. CNTs exhibit selective distribution in the PA phase, whereas CIM can form a relatively complete conductive path. The multi-flow provided by MFVIM can widen the distribution of conductive paths and increase conductivity by an order of magnitude of up to 5.1 × 10^−6^ S/m. Oriented conductive paths are enhanced by stable shear stress. However, oscillation shear stress provided by PVIM can tear conductive paths. Although the PA phase is oriented, it disperses, thereby destroying conductive paths and decreasing conductivity by six orders of magnitude.

## Figures and Tables

**Table 1:** Compositions and designation of HDPE/PA/CNT nanocomposites.

Sample	HDPE	PA	CNT
**A0.5(C/M/P)**	50 wt.%	50 wt.%	0.5 Phr
**A1(C/M/P)**	50 wt.%	50 wt.%	1 Phr
**A2(C/M/P)**	50 wt.%	50 wt.%	2 Phr
**B0.5(C/M/P)**	75 wt.%	25 wt.%	0.5 Phr
**B1(C/M/P)**	75 wt.%	25 wt.%	1 Phr
**B2(C/M/P)**	75 wt.%	25 wt.%	2 Phr

**Table 2:** Surface energy and polarities of the polymers and MWCNT.

Material	Temperature (°C)	Surface energy (mJ/m^2^)	Polarity (%)	Ref.
**CNT**	--	45.3	59	[48]
**PE**	240	23.2	0	[49]
**PE**	280	20.9	0	[49]
**PA**	280	32.2	30.1	[49]

## Supplementary Materials

The following are available online at https://www.mdpi.com/2073-4360/11/11/1779/s1, Figure S1: Selective stress-strain curves of different samples molded by (a) CIM, (b) MFVIM, and (c) PVIM.
