# Supplementary material for: Effects of Three Different Injection-Molding Methods on the Mechanical Properties and Electrical Conductivity of Carbon Nanotube/Polyethylene/Polyamide 6 Nanocomposite
Source: Polymers (Basel). 2019 Oct 30;11(11):1779. doi: 10.3390/polym11111779 (PMC6918180; doi:10.3390/polym11111779)
Supplement: Supplementary file 1 [file polymers-11-01779-s001.pdf]

polymers

Supplementary materials

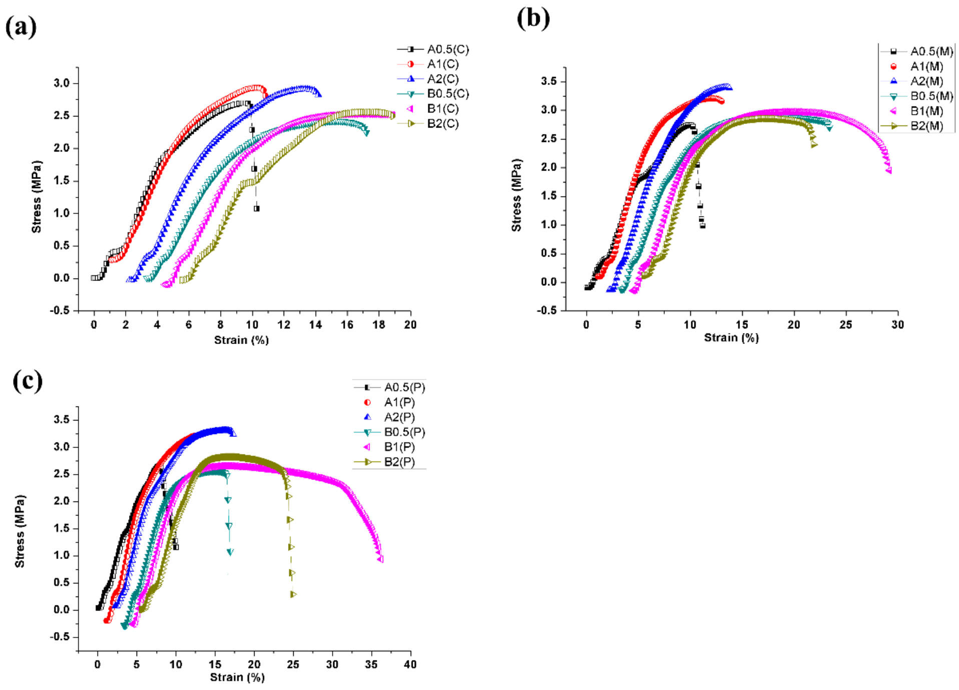

Figure S1. Selective stress-strain curves of different samples molded by (**a**) CIM, (**b**) MFVIM, and (**c**) PVIM.
